# Cross-sectional investigation of pathogenic *Leptospira* at the wildlife–livestock interface in a Cerrado–Atlantic Forest ecotone, Brazil

**DOI:** 10.1007/s11250-026-05345-7

**Published:** 2026-09-17

**Authors:** Rafael Quirino Moreira, Pollyanna Mafra Soares, Vanessa do Nascimento Ramos, Rick Anderson Freire Mangueira, Bruno Cabral Pires, Hugo Libonati de Araújo, Ana Carolina Guimarães Fenelon, Walter Lilenbaum, Anna Monteiro Correia Lima

**Affiliations:** 1 Laboratory of Infectious Diseases, Federal University of Uberlândia, Ceará Street, Block 2D, Room 33, Umuarama Campus, Uberlândia, MG 38405-315 Brazil; 2 Ixodology Laboratory, Federal University of Uberlândia, Pará avenue, Block 6T, Umuarama Campus, Uberlândia, MG 38405-320 Brazil; 3 Department of Statistics, Federal University of Campina Grande, Aprígio Veloso Street, 882, Universitário, Campina Grande, PB 58428-830 Brazil; 4 Laboratory of Veterinary Bacteriology, Biomedical Institute, Department of Microbiology and Parasitology, Fluminense Federal University, Prof. Hernani Melo Street, 101, São Domingos, Niterói, RJ 24210-130 Brazil

**Keywords:** Ecology, *Leptospira* spp., Transmission, Small mammals, Cattle

## Abstract

Leptospirosis is a globally distributed zoonosis affecting domestic and wild species, particularly prevalent in regions with high temperature and humidity that favor leptospiral survival in the environment. This study investigated the role of wildlife–livestock interactions in the transmission cycle of *Leptospira* spp. Serum samples (for Microscopic Agglutination Test – MAT, considering dilutions ≥ 1:100 with ≥ 50% agglutination as reactive) and kidney tissues (for *lipL32* PCR) were collected from 28 small mammals, including 14 marsupials and 14 rodents. All samples were seronegative by MAT; however, 15/28 (53.6%) kidney samples were PCR-positive, including 9/14 (64.3%) marsupials and 6/14 (42.9%) rodents. Additionally, 66 bovine serum samples from a dairy farm in the same area were analyzed by MAT, with 60/66 (90.9%) testing seropositive revealing high antibody titers (800), predominantly against serogroup Icterohaemorrhagiae (10%), followed by Sejroe (1.6%). Serogroup Pomona showed 1.6% of titers at 400. These findings suggest that small mammals might have played a significant role in the transmission and maintenance of pathogenic leptospires in the studied area, potentially acting as reservoirs and contributing to cattle infection. However, the observed infection patterns may also reflect indirect transmission routes, such as environmental persistence of leptospires in soil and water, or the involvement of additional, uninvestigated reservoir hosts within the agroecosystem. Therefore, small mammals sharing habitats with livestock should be considered important in leptospirosis epidemiology. Further studies evaluating leptospiral survival in environmental substrates, applying molecular typing to identify circulating strains, and conducting broader host surveillance are necessary to better elucidate transmission dynamics.

## Introduction

Leptospirosis is a zoonotic disease that occurs widely in regions with high temperatures and humidity, especially in Latin America and Southeast Asia. Its transmission occurs primarily among domestic, wild, and synanthropic animals through direct contact with infected animals or via exposure to water, soil, and food contaminated with the urine of animals infected with *Leptospira* spp. (Fornazari et al. 2018; Picardeau 2013).

The presence of small wild mammals in production and anthropized environments can contribute to pathogen exposure, thereby enabling increased transmission of pathogenic microorganisms (Simola et al. 2024). In Brazil, small mammals, primarily rodents and marsupials, comprise the largest number of species in the class Mammalia (Paglia et al. 2012). Several *Leptospira* spp. strains have been described in small mammals. The primary strains described in Brazil are members of serogroup Icterohaemorrhagiae, which have significant zoonotic importance and, in urban regions, are carried and disseminated by Norway rats (*Rattus norvergicus*) (Jorge et al. 2012; Cordeiro et al., 1981).

More than half of the southeastern part of the state of Goiás (located in the Cerrado biome) has been deforested owing to cattle-raising activities in this region (Agência Nacional de Águas, 2013; Cardoso et al. 2014). These anthropic changes cause a constant loss of habitats, which may affect ecological relationships. Within the “One Health” concept, monitoring the interactions between hosts, infectious agents, and the environment is essential (Jancloes et al. 2014).

Leptospirosis is an endemic disease and a constant threat to cattle in Southeast Goiás. Studies conducted by Marques et al. (2010) and Moreira et al. (2010) revealed high seroprevalence (54.1% and 81.1%, respectively) in bovine herds, primarily for the serogroup Sejroe. Therefore, owing to cattle-raising activities and the presence of small mammals in this region, the objective of this study was to investigate the interface between *Leptospira* spp. infection on wildlife and livestock in the leptospirosis transmission cycle.

## Methods

### Study design and setting

A cross-sectional observational study was conducted on a single cattle farm (approximately 68 ha) located in the municipality of Goiandira, southeastern Goiás, central Brazil (18.1630556 S; 48.1354722 W). The study was performed at the end of the rainy season and at the end of the dry season. The property consisted of pasture areas interspersed with native forest fragments characteristic of Cerrado formations influenced by the Atlantic Forest biome. Cattle had unrestricted access to forested areas and natural water sources. Water for animal consumption originated from an artesian well and the Mata da Fartura stream, a tributary of the Paranaíba River, without prior treatment. The animals ingested water from troughs and directly from the watercourse, and farm workers may come into contact with this water.

### Ethical approval

The procedures performed on the animals in this study were approved by the Ethics Committee on the Use of Animals (CEUA) of the Federal University of Uberlândia under protocol number 151/16, and by the Brazilian Environment Institute (IBAMA), through the Instituto Chico Mendes for Conservation and Biodiversity (ICMBio), under protocol number 52,983/1 in the Biodiversity Authorization and Information System (SISBIO).

### Study population and sampling strategy

#### Cattle

The target population consisted of all cattle present on the farm at the time of sampling. A total of 66 animals (Gir and crossbred Holstein-Gir breeds) were sampled, including one bull, 46 adult females (> 48 months old), seven steers, and 12 heifers (6–24 months old). Sampling was conducted by convenience, as all accessible animals during routine handling were included. No formal sample size calculation was performed, as the objective was to assess herd-level exposure within a single property. The unit of analysis for cattle was the individual animal.

#### Small mammals

**Fig. 1:**
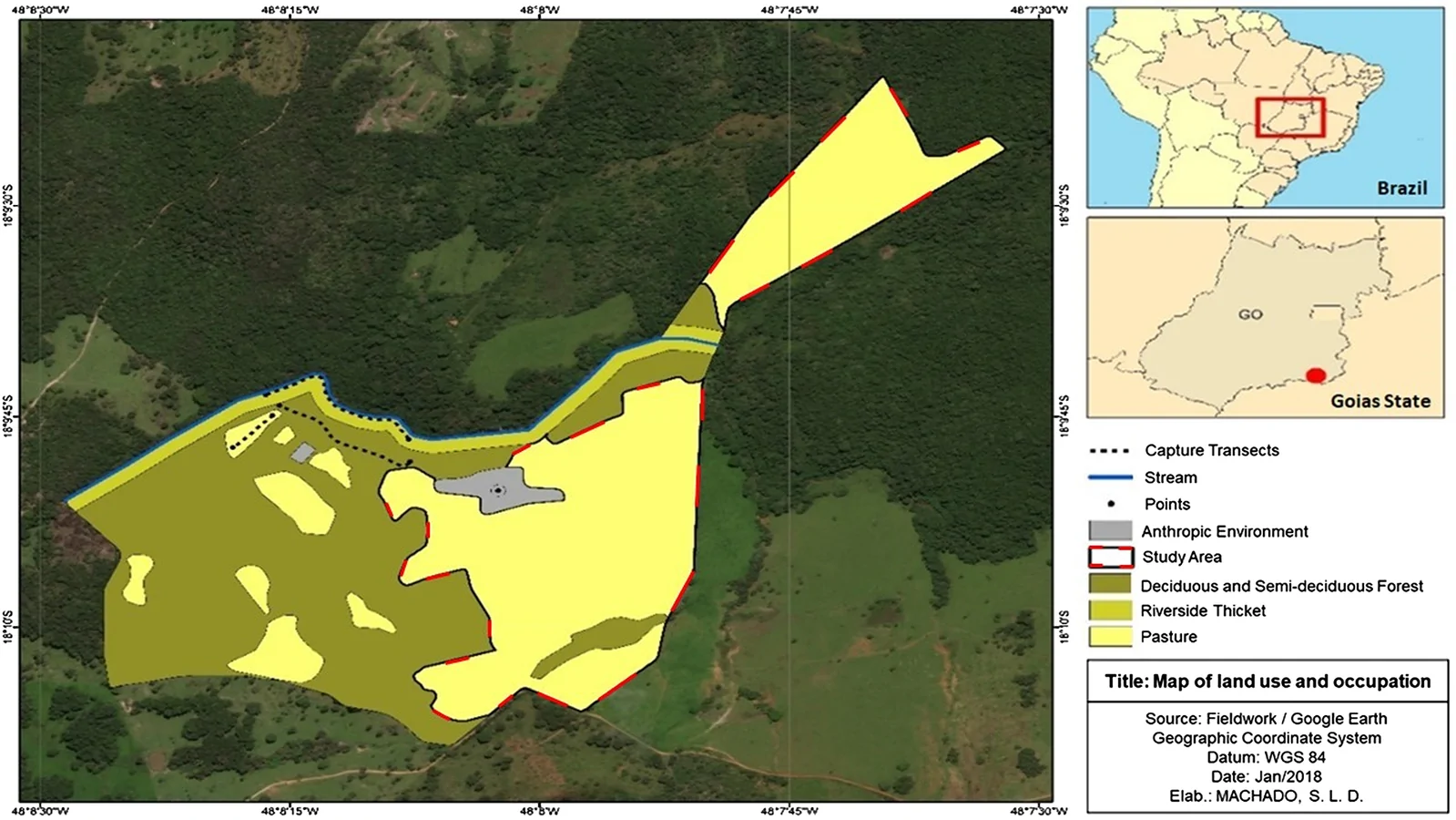
Map representing the use, land occupation, and capture transects of small non-flying mammals, located in the municipality of Goiandira - Goiás, Brazil

Small mammals were captured in four predefined environmental strata within the property (forest fragments, pasture, riparian vegetation, and transitional areas). Two trapping campaigns of four consecutive nights each were conducted, one during the rainy season and one during the dry season, as described in Table 1 and shown in Fig. 1. Sherman traps were used in (1), (2), and (3), and Sherman and Tomahawk traps were used in (4). Trapping locations were selected to represent habitat heterogeneity rather than through probabilistic sampling.

**Table 1:** Description of the four different environments on the property of the study area where the capture of small mammals was carried. Goiandira – GO, Brazil

Environment	Description area
(1) Anthropic environment	places close to human dwellings, storehouse, engine rooms, storage facilities for inputs
(2) Deciduous and semi-deciduous forest	formations composed of arboreal stratum that lose all or part of the leaves during the dry season, according to the classification of Oliveira-Filho and Ratter in 2002
(3) Riverside thicket	semi-deciduous or deciduous seasonal thicket that accompany the Ribeirão Mata da Fartura margin, according to the classification of Oliveira-Filho and Ratter in 2002
(4) Cattle grazing pasture	cattle grazing area, bordered by some of the thicket previously described, with the predominance of *Brachiaria decumbens* grass at its maximum growth height

Captured animals were identified to the species level, sexed, and measured. Pregnant or lactating females were released after recovery, according to the protocol used by Silva (2014). Other animals were euthanized according to national guidelines by intracardiac exsanguination (CONCEA, 2022; CFMV, 2013).

Some animals (in pregnant or lactating) were subjected to blood collection in the lateral tail vein, whereas others were euthanized according to pre-established protocols. Blood serum was stored at − 20 °C until analysis (CONCEA, 2022; CFMV, 2013; Moreira et al. 2010).

From the euthanized specimens, in addition to blood, the kidneys were collected, placed in identified sterile tubes, frozen at − 80 °C, and subsequently used for polymerase chain reaction (PCR).

### Laboratory procedures

#### Serology

Blood serum samples from cattle and small mammals were sent to the Laboratory of Infectious Diseases of the Faculty of Veterinary Medicine of the Federal University of Uberlândia and tested using the microscopic agglutination test (MAT) for antibodies against 16 serogroups of *Leptospira* spp., comprising 22 serovars (21 reference strains and four Brazilian field strains) as described in Table 2.

**Table 2:** Antigens used in the microscopic agglutination test (MAT), according to strain, species, serogroup, and serovar

Standard Strains			
Serogroup	Species	Serovar	Strain
Australis	*L. interrogans*	Australis	Ballico
	*L. interrogans*	Bratislava	Jez Bratislava
Autumnalis	*L. interrogans*	Autumnalis	Akiyami
Bataviae	*L. interrogans*	Bataviae	Van Tienen
Ballum	*L. borgpetersenii*	Castellonis	Castellon 3
Canicola	*L. interrogans*	Canicola	Hond Utrecht IV
Cynopteri	*L. kirschneri*	Cynopteri	3522 C
Djasiman	*L. interrogans*	Djasiman	Djasiman
Grippotyphosa	*L. kirschneri*	Grippotyphosa	Moskva V
Hebdomadis	*L.interrogans*	Hebdomadis	Hebdomadis
Icterohaemorrhagiae	*L. interrogans*	Copenhageni	M20
	*L. interrogans*	Icterohaemorrhagiae	Verdun
Javanica	*L. borgpetersenii*	Javanica	Poi
Panama	*L. noguchi*	Panama	CZ 214k
Sejroe	*L. santarosai*	Guaricura	BOV G
	*L. borgpeterseni*	Hardjobovis	Sponselee
	*L. interrogans*	Hardjoprajitno	Hardjoprajitno
	*L. borgpetersenii*	Sejroe	M84
	*L. interrogans*	Wolffi	3705
Shermani	*L. santarosai*	Shermani	1342 K
Tarassovi	*L. borgpetersenii*	Tarassovi	Perepelitsin
**Strains of Isolates in Brazil (small mammals)**			
**Serogroup**	**Species**	**Serovar**	**Strain**
Bataviae	*L. santarosai*	Brasiliensis	AN776
Icterohaemorrhagiae	*L. interrogans*	Copenhageni	M10/99
	*L. interrogans*	Copenhageni	Rato
Pomona	*L. kirschneri*	Uncharacterized	M36/05

The collections of standard and local strains in Brazil used for serological diagnosis were donated by the Veterinary Bacteriology Laboratory of the Department of Microbiology and Parasitology at the Federal Fluminense University. Samples were considered reactive at ≥ 50% agglutination at dilutions from 1:100 to 1:3200 (Lee and Goosens 2015; Paixão et al. 2014; Adler and Moctezuma 2010; Marques et al. 2010). MAT results were interpreted considering the known cross-reactivity between serogroups.

#### Polymerase chain reaction (PCR)

Kidney tissue from euthanized small mammals was subjected to DNA extraction using the DNeasy^®^ Blood & Tissue Kit (Qiagen). Polymerase Chain Reaction (PCR) targeting the *lipL32* gene (243 bp fragment) was performed using previously described primers, *lipL32*-45 F (5′-AAG CAT TAC CGC TTG TGG TG-3′) and *lipL32*-286R (5′-GAA CTC CCA TTT CAG CGA TT-3′), based on the method of Stoddard et al. (2009), to amplify a 243 bp fragment.

PCR was performed in a final volume of 25 µL containing 3.5 mM MgCl2, 7.5 pmol of each primer, 1 × GoTaq ^®^ Reaction Buffer, 250 µM of each deoxynucleotide triphosphate (dNTP), 0.5 U of GoTaq^®^ DNA Polymerase (Promega), and 6 µL of DNA. The amplification protocol consisted of initial denaturation at 94 °C for 2 min, 40 cycles at 94 °C for 30 s, 52 °C for 30 s, and 72 °C for 1 min, and final extension at 72 °C for 5 min. As a positive control, the standard strain *Leptospira* interrogans serovar Copenhageni strain FIOCRUZ L1-130 was used.

Finally, for the verification of the PCR, the electrophoresis method in 2% agarose gel with 0.5 X TBE buffer (Tris, boric acid, and ethylenediaminetetraacetic acid [EDTA]) was performed, with the DNA present in the PCR products stained with a 20X solution Gel Red^®^ (Biotium) and visualized under ultraviolet light.

The detection of *lipL32* indicated the presence of pathogenic *Leptospira* DNA but not necessarily active shedding.

### Bias and limitations

Potential sources of bias include convenience sampling of cattle, non-probabilistic wildlife trapping, limited sample size in certain strata, potential misclassification owing to MAT cross-reactivity, absence of culture or sequencing to confirm strain identity, and the cross-sectional design, which precludes inference of temporality and transmission directionality.

### Statistical analysis

Apparent prevalence and PCR positivity were calculated as proportions with 95% exact binomial confidence intervals (CIs, Clopper–Pearson method). Associations between categorical variables were assessed using Pearson’s chi-squared test when assumptions were met (expected counts ≥ 5). Fisher’s exact test was used when small, expected frequencies were observed. All analyses were performed using R software (R Core Team, 2016). Statistical significance was set at *P* < 0.05.

## Results

Of the 66 bovine serum samples analyzed, 60 were seropositive, resulting in an apparent seroprevalence of 90.9% (60/66; 95% CI: 81.3–96.6%, exact binomial). Antibody titers ranged from 100 to 800, predominantly against the serogroups Icterohaemorrhagiae (37/60; 61.7%; 95% CI: 48.2–73.9%), Pomona (11/60; 18.3%; 95% CI: 9.5–30.4%), Sejroe (6/60; 10.0%; 95% CI: 3.8–20.5%), Tarassovi (4/60; 6.7%; 95% CI: 1.8–16.2%), Djasiman (1/60; 1.7%; 95% CI: 0.04–8.9%), and Javanica (1/60; 1.7%; 95% CI: 0.04–8.9%), as shown in Table 3.

**Table 3:** Frequency of anti-Leptospira agglutinins by serogroup of bovines (MAT) from Goiandira – GO, Brazil

Serogroups	Titers frequency (*n*/%)			
	100	200	400	800
Icterohaemorrhagiae	16 (26.6)	10 (16.6)	05 (8.3)	06 (10)
Sejroe	03 (5)	02 (3.3)	--	01 (1.6)
Pomona	10 (16.6)	--	01 (1.6)	--
Tarassovi	01 (1.6)	01 (1.6)	02 (3.3)	--
Djasiman	01 (1.6)	--	--	--
Javanica	01 (1.6)	--	--	--
**Total**	32 (53.3)	13 (21.6)	8 (13.3)	7 (11.6)

The highest antibody titers were detected for serogroups Icterohaemorrhagiae and Sejroe (*n* = 7; 11.6%), with titers reaching 800; eight animals (13.3%) showed antibody titers of 400 for serogroups Icterohaemorrhagiae, Pomona, and Tarassovi; and 13 (21.6%) showed antibody titers of 200 for serogroups Icterohaemorrhagiae, Sejroe, and Tarassovi (Table 3). The other reactive serogroups in this study, such as Djasiman and Javanica, presented titers of 100.

During the two small mammal capture campaigns, 28 small mammal specimens were captured. Of these, 14 were marsupials (*Gracilinamus agilis)*; the others were five rodent species (*Calomys tener*, *n* = 2; *Hylaeamys megacephalus*, *n* = 5; *Nectomys squamipes*, *n* = 1; *Oecomis bicolor*, *n* = 3; *Rattus rattus*, *n* = 3). Additionally, three females (*Gracilinanus agilis*, *n* = 1; *Oecomis bicolor*, *n* = 1; *Hylaeamys megacephalus*, *n* = 1) were captured.

None of the serum samples collected from the captured specimens (rodents and marsupials) were reactive to MAT; in contrast, 15/28 kidney samples were positive (53.6%; 95% CI: 33.9–72.5%) in the PCR for the *lipL*32 gene, of which nine (64.3%; 95% CI: 35.1–87.2%) specimens of *Gracilinanus agilis* and six (42.9%; 95% CI: 17.7–71.1%) rodents (*Calomys tener*, *n* = 2; *Hylaeamys megacephalus*, *n* = 2; *Oecomys bicolor*, *n* = 1; *Nectomys squamipes*, *n* = 1), as shown in Table 4.

**Table 4:** Small non-flying mammals captured and the number of PCR reagents for the lipL32 gene of pathogenic leptospires from DNA extracted of renal tissue, in their respective capture environments and trap installation locations. Goiandira - GO, Brazil

	Total specimens captured		Capture environments								Trap installation location			
			Anthropic		Cattle grazing pasture		Deciduous and semi-deciduous forest		Riverside thicket		Soil		Tree	
	*N*	PCR (*n*/%)	*N*	PCR (*n*/%)	*N*	PCR (*n*/%)	*N*	PCR (*n*/%)	*N*	PCR (*n*/%)	*N*	PCR (*n*/%)	*N*	PCR (*n*/%)
Marsupials	14	9 (64.3)	0	0	0	0	11	8 (72.7)	3	1 (33.3)	6	5 (83.3)	8	4 (50)
Rodents	14	6 (42.9)	3	0	3	2 (66.6)	2	1 (50)	6	3 (50)	11	5 (45.4)	3	1 (33.3)
Total	28	15 (53.6)	3	0	3	2 (66.6)	13	9 (69.2)	9	4 (44.4)	17	10 (58.8)	11	5 (45.4)

At the end of the dry season, 23 small mammals were captured (13 marsupials and ten rodents), of which 14 (nine marsupials and five rodents) tested positive by PCR, corresponding to a positivity rate of 60.9% (14/23; 95% CI: 38.5–80.3%). At the end of the rainy season, five small mammals were captured (four rodents and one marsupial), and only one rodent (*Hylaeamys megacephalus*) tested positive by PCR, resulting in a positivity rate of 20.0% (1/5; 95% CI: 0.5–71.6%) (Table 5). No statistically significant association was detected between PCR positivity and taxonomic order or trapping season. However, given the small sample size, particularly during the rainy season, and the low expected cell counts in the contingency tables, these results and comparisons should be interpreted with caution.

**Table 5:** Small mammals captured and the number of PCR reagents for the lipL32 gene of pathogenic leptospires from DNA extracted of renal tissue, in their respective capture campaigns (dry and rainy season). Goiandira – GO, Brazil

	Total of animals captured		Species	Seasons			
				Rainy		Dry	
	*N*	PCR (*n*/%)		*N*	PCR (*n*/%)	*N*	PCR (*n*/%)
Marsupials	14	9 (64.3)	*Gracilinamus agilis*	1	0	13	9(69.2)
Rodents	14	6 (42.9)	*Calomys tener*	0	0	2	2 (100)
			*Hylaeamys megacephalus*	2	1 (50)	3	1 (33.3)
			*Nectomys squamipes*	0	0	1	1 (100)
			*Oecomys bicolor*	1	0	2	1 (50)
			*Rattus rattus*	1	0	2	0
Total	28	15 (53.6)		5	1(20)	23	14(60.9)

## Discussion

The high seroprevalence observed in the studied bovine herd (90.9%) suggests an enzootic pattern of exposure to pathogenic *Leptospira*, as supported by a similar prevalence rate (87.2%) reported by Moreira et al. (2010) in the same region, particularly in the absence of implemented control measures. The elevated seroprevalence observed in cattle is consistent with previous reports from the same region, supporting the hypothesis of endemic exposure. However, given the cross-sectional design, temporal dynamics and directionality of transmission cannot be inferred.

Several associated factors might have contributed to the elevated prevalence of bovine infection observed in this study, including the presence of wildlife sharing the same environment and the use of untreated river or well water for animal watering, which may increase the risk of exposure compared with chlorinated water sources (Paixão et al. 2014; Wynwood et al. 2014; Cilia et al. 2021). Furthermore, Thibeaux et al. (2017) revealed that soil can provide a protective environment that enables the persistence of leptospires, acting as an environmental reservoir and maintaining their viability for months.

Seasonal variation in PCR positivity was observed descriptively, with a higher proportion of infected small mammals detected during the dry season compared to the rainy season. This pattern may reflect ecological and behavioral adaptations, as reduced water availability during the dry period can concentrate animals around limited water sources, increasing contact rates between individuals and environmental contamination with leptospires (Wynwood et al. 2014; Cilia et al. 2021). In addition, scarcity of food resources during the dry season may drive small mammals to forage more intensively in cattle-associated environments, thereby increasing the likelihood of interspecies exposure (Thibeaux et al. 2017).

Conversely, during the rainy season, although environmental conditions such as increased humidity and soil moisture may enhance leptospiral survival, the greater spatial dispersion of hosts and dilution effects in water bodies may reduce the probability of detecting infected individuals within the sampled population (Wynwood et al. 2014). Therefore, the observed seasonal pattern likely reflects a combination of ecological dynamics, host behavior, and environmental persistence of the pathogen.

It is important to note that the limited number of animals captured during the rainy season restricts the robustness of seasonal comparisons, and these findings should be interpreted as exploratory rather than confirmatory.

Although rodents are traditionally considered primary maintenance hosts of pathogenic leptospires, marsupials also showed substantial PCR positivity in the present study. The absence of MAT reactivity in small mammals, despite PCR positivity, may reflect chronic renal carriage with low or undetectable circulating antibody titers, a pattern previously described in maintenance hosts. Rodents and marsupials are known to be important reservoirs of pathogenic leptospires, as they remain infected for a long period without manifesting the disease (Adler and Moctezuma 2010). However, the Rodentia order is considered the primary reservoir of this pathogen in the environment (Thibeaux et al. 2017; Nakamura et al. 2013; Adler and Moctezuma 2010).

In addition, the limited sensitivity of MAT and its dependence on the panel of reference serovars may result in failure to detect antibodies against locally circulating or uncharacterized strains, which could also contribute to the absence of serological reactivity observed in small mammals.

In a study conducted by Cutolo et al. (2020), wild animals (rodents and marsupials) found by residents in anthropized areas (rural, peri-urban, and urban) in the municipality of Monte Mor showed reactivity in the MAT test for serogroup Icterohaemorrhagiae.

Medeiros et al. (2020) in the western Brazilian Amazon and Rocha et al. (2020) in the eastern Brazilian Amazon reported the first evidence of *Leptospira* spp. carrier/hosts based on the high prevalence of pathogenic *Leptospira* spp. detected in rodent and marsupial species through PCR performed on kidney samples, followed by sequencing of positive samples, which identified *Leptospira interrogans* as the most frequent species.

In this study, the captured small mammals (rodents and marsupials) did not show agglutinins against *Leptospira* spp.; however, PCR showed a high infection rate. Hosts that are considered natural reservoirs of *Leptospira* spp., due to evolutionary adaptation to infection by this bacterium, may have undetectable levels of antibodies in the blood serum, with concentrations lower than those widely accepted, such as a dilution of 1:100 (Ellis 2015).

Thus, these findings indicate that the six small mammal species in this study may be important renal carriers of pathogenic *Leptospira* and participate in the shedding of the bacterium in the environment. Similar findings have been reported in other regions of Brazil, including the Pantanal (Vieira et al. 2016), the municipality areas in the municipality of Botucatu (Fornazari et al. 2018), the Brazilian Amazon (Medeiros et al. 2020; Rocha et al. 2020), and the central region of São Paulo (Silva et al. 2023).

Because of the low sensitivity of MAT in the identification of natural reservoirs of leptospires (rodents and marsupials), we sought to detect the serogroups of pathogenic leptospires circulating in the environment of the study through cattle kept in the same area as these natural reservoirs. Under these circumstances, a higher frequency and higher titers of MAT in cattle seroreactive to the serogroup Icterohaemorrhagiae were observed for the standard strain (serovar Icterohaemorrhagiae), as well as for strains M10/99 and Rat (serovar Copenhageni) isolated from rodents.

Ellis (2015) described that serogroup Icterohaemorrhagiae causes incidental infection in cattle owing to contact with an environment contaminated by the urine of their reservoirs; in addition, Esteves et al. (2005) and Ullmann and Langoni (2011) showed that animals in the same environment mutually infect each other with their serogroups. The predominance of the Icterohaemorrhagiae serogroup in cattle may be epidemiologically relevant, as this serogroup is commonly associated with incidental infection in bovines following environmental contamination by reservoir hosts. Nevertheless, direct epidemiological linkage cannot be confirmed without molecular typing or strain comparison between host species.

Additional environmental parameters may also influence infection patterns in this system. The presence of untreated water sources, combined with soil characteristics and vegetation cover, may create microhabitats favorable for leptospiral persistence (Thibeaux et al. 2017). Furthermore, anthropogenic landscape modification, including deforestation and pasture expansion, may alter host community structure and increase overlap between wildlife and livestock, thereby facilitating pathogen circulation (Cilia et al. 2021).

Landscape modification and anthropogenic environmental changes may influence wildlife–livestock interactions and pathogen circulation. However, causal relationships cannot be established within the framework of this cross-sectional design.

In this study, all six species of small wild mammals captured were identified as renal carriers of pathogenic leptospires, predominantly during the dry season. This pattern may be associated with reduced availability of food and water, leading these mammals to seek resources in cattle production environments, thereby increasing opportunities for the circulation of pathogenic *Leptospira* species between wildlife and livestock. This suggests the wide variety of hosts for *Leptospira* spp. and indicates that this pathogen is well adapted to different species of small wild mammals in the Cerrado–Atlantic Forest ecotone environment, as reported by Aliberti et al. (2022) in Northeastern Sicily and Harran et al. (2024) in France.

In forested and cattle-grazing capture environments, small wild mammals carrying pathogenic leptospires in the kidneys were detected, with 69% of positive detections occurring in specimens captured in deciduous and semi-deciduous forest areas, followed by cattle-grazing pastures (66%) and riverside thickets (44%). These findings indicate the contribution of small wild mammals to the maintenance and environmental dissemination of pathogenic *Leptospira* spp. within the studied property and indicate the circulation of these bacteria across different habitats, as supported by the diversity of serogroups identified through MAT-based serological testing of cattle.

Among the captured species, only one belonged to the order Marsupialia (*Gracilinanus agilis*), which showed a higher proportion of renal carriage of pathogenic leptospires (64.3%) than rodents (42.9%). These findings are consistent with those reported by Cilia et al. (2021), who demonstrated that a wide diversity of *Leptospira*spp. can be isolated from opossum kidney and urine. Opossums are predominantly arboreal mammals (Cordeiro et al. 1981) and, in this study, were captured exclusively in forested environments, particularly deciduous and semideciduous forests, where eight of 11 specimens (72.7%) were PCR-positive, with most detections occurring toward the end of the dry season (69.2%).

In the environment of degraded pasture, of the three captured rodents (*Calomys tener*), two were positive for the *lipL*32 gene in the DNA extracted from renal tissues. Importantly, the detection of the *lipL32* gene in kidney tissue indicates the presence of pathogenic *Leptospira* DNA but does not confirm active shedding or transmission capacity. Therefore, although these species may act as renal carriers, this study does not establish their definitive role as maintenance reservoirs without longitudinal or culture-based evidence. Seasonal differences in PCR detection were observed descriptively, with higher positivity during the dry season. However, the limited sample size, particularly during the rainy season, restricts robust inferences regarding seasonal patterns.

A representative of the species *Nectomys squamipes* (positive for PCR) was also identified in the capture environments; this species becomes more susceptible to contact with contaminated water through the urine of other animals harboring renal *Leptospira* spp. (Ko et al. 2009; Martins et al. 2006; Cordeiro et al. 1981).

In this study, 53.57% of small wild mammals were identified as renal carriers of pathogenic *Leptospira* spp., and 90.9% of cattle were reactive, as shown using the MAT. These findings might be associated with deforestation and landscape modification in anthropized and pasture environments for livestock development, leading to ecological imbalance in these and adjacent areas and favoring the transmission of pathogenic *Leptospira* spp. to cattle, predominantly strains belonging to the Icterohaemorrhagiae serogroup.

Finally, because the serogroup Icterohaemorrhagiae is recognized as pathogenic in humans, occupational exposure of farm workers should be considered a potential public health concern, although human infection was not evaluated in this study. Since the human species is an accidental host susceptible to infection by *Leptospira* spp. (Ko et al. 2009).

## Conclusion

This study, conducted at the wildlife–livestock interface in an environment altered by anthropogenic activities, identified an extremely high seroprevalence in cattle alongside the seronegative carriage of *Leptospira* in all wild species investigated. These findings highlight the possibility that wildlife and livestock amplify exposure to and the circulation of this bacterium in this agroecosystem. Future studies using molecular typing to identify circulating strains and assess leptospiral survival in soil and water, as well as comprehensive host surveillance are required to clarify transmission dynamics and the role of anthropogenic activities in the ecology of leptospirosis. In addition, the public health risk posed by pathogenic *Leptospira* in these species and in this type of environment should be considered and further researched.

## Data Availability

The datasets generated during and/or analysed during the current study are not publicly available due to for the author to wait for publication but are available from the corresponding author on reasonable request.
